# Effect of Adjunctive Topical Liposomal Azithromycin on Systemic Azithromycin on Old World Cutaneous Leishmaniasis: A Pilot Clinical Study

**DOI:** 10.22037/ijpr.2020.113710.14445

**Published:** 2021

**Authors:** Bahareh Abtahi-Naeini, Sajjad Hadian, Fatemeh Sokhanvari, Amirali Hariri, Jaleh Varshosaz, Zabihollah Shahmoradi, Awat Feizi, Farzin Khorvash, Atousa Hakamifard

**Affiliations:** a *Skin Diseases and Leishmaniasis Research Center, Isfahan University of Medical Sciences, Isfahan, Iran. *; b *Department of Infectious Diseases, School of Medicine, Isfahan University of Medical Sciences, Isfahan, ran. *; c *Department of Pharmaceutics, School of Pharmacy and Pharmaceutical Sciences, Isfahan University of Medical Sciences, Isfahan, Iran. *; d *Department of Pharmaceutics, Drug Delivery System Research Center, School of Pharmacy, Isfahan University of Medical Sciences, Isfahan, Iran. *; e *Department of Biostatistics and Epidemiology, School of Health, Isfahan University of Medical Sciences, Isfahan, Iran* *. *; f *Acquired Immunodeficiency Research Center, Isfahan University of Medical Sciences, Isfahan, Iran. *; g *Infectious Diseases and Tropical Medicine Research Center, Shahid Beheshti University of Medical Sciences, Tehran, Iran. *

**Keywords:** Azithromycin, Leishmaniasis, Liposomes, Macrolides, Topical administration

## Abstract

The treatment of Cutaneous Leishmaniasis (CL) is complex, and the search for safer, more efficient, and cost-effective treatments is ongoing. This study aimed to evaluate the efficacy of the combination of liposomal and oral azithromycin as the first clinical study against CL. This assessor-blind, randomized clinical trial was conducted in out-patients Leishmaniasis clinic of Skin Diseases and Leishmaniasis. The cutaneous lesions of eligible participants were randomized to receive either oral azithromycin or the combined oral and topical liposomal azithromycin. All participants received 250 mg of azithromycin twice daily or 8 mg/per kg for 4 weeks. In the combination group, a topical liposomal formulation of 0.04 mmol/mL of azithromycin was administered as 0.2-0.5 cc twice daily according to the lesion size in order to make a thin layer of the drug on the surface of the lesion. The size and induration changes from baseline to the end of the study were analyzed. Twenty-one lesions of 13 patients in the combination group and 20 lesions of 14 patients in the oral group were recruited. The mean ± SD of improvement was significantly different between two groups after 12 weeks (3.89 ± 0.46 *vs.* 3.15 ± 1.23 *P *= 0.02 combination group *vs.* oral group respectively). The patients did not experience any systemic adverse effects related to azithromycin and the only adverse effects related to topical treatment were mild pruritus in 2 cases. In conclusion, the combination of oral and topical liposomal formulation of azithromycin is safe and effective to treat CL.

## Introduction

The treatment of Cutaneous Leishma-niasis (CL) is complex (1, 2), although meglumine antimoniate is the first-choice treatment for CL (3). However, inconsistent results and significant side effects have not received FDA approval yet (4).

Today there is no ideal, available therapy for CL and its treatment remains a challenge (1, 2), and the search for safer, more efficient, and cost-effective treatments is ongoing. 

The treatment of CL should be individualized and factors, such as extent and location of lesions, patient comorbidities, and previous treatments patient wishes, need to be included in individual risk-benefit treatment decisions (1).

Azithromycin is one of the macrolide deriv-atives structurally related to erythromycin. Its anti-leishmaniasis activity has been reported in some in vivo and *in-vitro* studies (5-7).

Its oral administration, the long half-life, and its safety in children and pregnancy are advantages for treating leishmaniasis with azithromycin. Although azithromycin seems to be an appropriate alternative to treat CL, the clinical results of azithromycin are variable (5, 8-11). 

This issue illustrates the need for a controlled clinical trial to evaluate the efficacy of azithromycin in different ways of administration (oral *vs.* topical) in the treatment of different populations (adult *vs.* children) affected by CL.

Liposomes are hollow spheres of lipid bilayers, mainly consisting of phospholipids and are widely used as the carriers of active ingredients to human tissues and also as lipid transfer vesicles to the skin, so developing the use of liposomal formulations as an optimum technically and the clinically feasible topical product is more preferred (12, 13).

Topical azithromycin is safe and the most frequently reported adverse are mild to moderate skin reactions, in most cases (14). 

Recently, liposomal azithromycin has been shown as an effective treatment for leishmaniasis in clinical studies (15). The combination of two drugs or the combination of local therapy with systemic therapy can be an alternative to increase the efficacy of local therapy. Some studies have evaluated the efficacy of this type of combination to treat CL with positive results (16, 17). 

Considering the anti-leishmaniasis activity of azithromycin and also the higher efficacy of liposomal drugs and combinational therapy, we decided to evaluate the efficacy of the combination of liposomal and oral azithromycin as a first clinical study of anti-leishmaniasis effects of the combination of oral and topical liposomal azithromycin against CL.

## Experimental

*Trial design and Participants*

From September 2018 to September 2019, a single-center, randomized, open-label, parallel-group trial was performed in the leishmaniosis outpatient clinic of Skin Diseases and Leishmaniasis Research Center, a referral center for cutaneous leishmaniasis affiliated to Isfahan University of Medical Sciences, Isfahan, Iran). The trial was approved by the ethics committee of Isfahan University of Medical Science (Grant No: IR.MUI.RESEARCH.REC.1397.144). It was conducted according to the Declaration of Helsinki and subsequent revisions and was registered at the Iranian of clinical trials (www.irct.ir; unique registration number: IRCT20180425039414N1). The written informed consent was previously obtained from all patients.

New Patients aged above one year, with a confirmed diagnosis of CL, either a direct smear stained with Giemsa, PCR or skin biopsy, were screened for the study. Subjects with less than 5 lesions and less than 5 centimeters in diameters were included. Patients were excluded from the study if they had 1- lesions on joint or a mucus membrane, sporotrichoid pattern, 2- pregnancy, 3- breast-feeding, 4- taking any other specific treatment for CL while participating in the study, 5- taking any medicatins that interfere with azithromycin while participating in the study, 6- any contraindication for the use of azithromycin,7- any hypersensitivity to macrolide antibiotics or ketolide antibiotics, 8-a significant medical underlying disease such as cardiac, renal, or liver dysfunction.

The following withdrawal criteria were applied: not showing up for follow-up visits, not taking the medication according to the study protocol, receiving other topical or immunosuppressive agents during the study, and non-tolerable side effects.

*Treatment protocol*

The cutaneous lesions of eligible participants were randomized to receive either oral azithromycin (Oral group) or combined oral and topical liposomal azithromycin (Oral + liposomal group).

We used Random allocation software for parallel-group randomized trials introduced by Saghaei (18). Regardless of group assignment of the lesions, all participants received 250 mg of azithromycin (Azithromycin FARABI 250 mg oral tablet, Farabi Pharmaceutical CO.) twice daily or 8 mg/per kg for 4 weeks.

In the Oral + liposomal group, liposomal azithromycin was administered as 0.2- 0.5 mL (6 to 15 mg) twice daily according to the lesion size in order to make a thin layer of the drug on the surface of the lesion.

Powder of azithromycin was kindly provided by Farabi Pharmaceutical Co., Iran.

The liposomes were prepared by a hydration-dehydration method. For this purpose, 114 mg of dipalmitoylphosphatidylcholine (DPPC) and 10 mg of cholesterol (molar ratio of DPPC to cholesterol was 6:1) were dissolved in an adequate mixture of chloroform/methanol (2:1) in a round bottle, attached to the rotary evaporator for complete drying and production a thin film. Then 30 mg of azithromycin was dissolved in phosphate buffer solution (PBS) (pH 7.4). The obtained aqueous solution was used for the hydration of the lipid thin film. The obtained suspension was vortexed for 2 min (45 s on and 10 s off cycles) in an ultrasonic bath (POWER-SONIC 505, Korea) under 45 Hz frequency of ultrasound waves. Then the dispersion was freeze-dried (Christ Alpha 4.2LD over, Germany) and kept in the refrigerator. The operating conditions of the freeze-drying were at the temperature of −40 °C and a 0.4 bar pressure.  For rehydration of the freeze-dried powder 100 μL of PBS was added and vortexed at 40 °C for 5 min. This was repeated 3 times and at last, by adding 700 μL of PBS, the final volume was adjusted on 1 mL. The final product contained 0.04 mmol/mL (30 mg/mL) of azithromycin.

*Outcome assessment*

The main outcome measure was the difference in lesion size change (the extent of re-epithelialization in ulcerative lesion) and lesion induration from the baseline to the 2 and 6 weeks after the termination of treatment period between the two groups. The treatment period was 4 weeks for each group and the patients were followed up weekly during the treatment course and 2 and 6 after that. 

The patients have also studied once again, 6 months after termination of the treatment course. The therapeutic results were categorized as follows: No improvement, partial improvement, complete improvement. 

Complete improvement: full re-epithelialization of the lesions for ulcerative ones or disappearance of induration and erythema; Partial improvement were classified as follows: 

(a) Slight improvement: decrease in size up to 25%,

(b) Mild: decrease in size between 25 and 50%,

(c) Moderate improvement: decrease in size between 50 and 75%,

(d) Significant improvement: decrease in size more than 75%.

Photographs of pre-and post-treatment were evaluated by two dermatologists who were blinded to the type of treatments.

*Statistical analysis*

Data analysis was conducted using SPSS version 24; IBM Company, USA. 

Numerical variables were summarized using mean ± SD and categorical variables presented as a number of patients and percentages. For evaluation of Global improvement, each improvement state is defined as an ordinal number; Complete improvement: 4, Significant improvement: 3, Moderate improvement: 2, Mild improvement: 1 and Slight improvement: 0. The mean difference between the two groups was reported as mean difference [MD, 95% confidence interval (CI)]. Two-factor repeated measure ANOVA was used to evaluate time treatment interaction between the treatment groups. Chi-square or Fisher’s exact tests were used to compare proportions between the two groups as appropriate. *P* < 0.05 was considered as significant.

## Results

In this clinical trial, 21 lesions of 13 patients in the combination group and 20 lesions of 14 patients in the oral group were recruited. There was no significant baseline difference between the two groups in terms of age (*P* = 0.84), sex (*P* = 0.85) and location of lesions (*P *= 0.33) (Table 1). Similarly, there was no significant difference in frequency of the type of lesions between the two groups at baseline (*P* > 0.05). Demographics and disease characteristics before the initiation of treatment in two groups were summarized in **(**Table 1). 21 lesions were treated with oral + liposomal azithromycin, and 20 lesions were treated with oral azithromycin, 20 lesions in the combination group and 18 lesions in the oral azithromycin group completed the study. The clinical evaluation of the patients regarding the induration at the 12^th^ week of the study showed statistically significant differences between before and after in both groups (Table 2). Using the Mann-Whitney U test for between-group analyses, there were marginally significant differences (*P* = 0.09) (Table 2). Lesion size changes between two groups and within-group were summarized in (Table 3). Distributions of Improvement of each group were summarized in (Table 4). The mean+/- SD of Improvement was significantly different between the two groups after the 12^th^ of the study (3.89 ± 0.46 *vs.* 3.15 ± 1.23 *P *= 0.02 combination group vs. oral group, respectively). No patient experienced systemic adverse effects related to azithromycin and the only adverse effects related to topical treatment were mild pruritus in 2 cases.

*Safety assessment*

Any signs or symptoms of skin reactions, including pruritus, burning, skin redness, edema, and scaling after using the formulation, were recorded. None of the patients showed any signs of allergy or inflammation of the skin until the end of the treatment period and after that, there were no complaints of inflammation and any skin problems.

**Table 1:** Characteristics of the patients in the two treatment groups

**Group**		**Oral + liposomal group**	**Oral group**	***P-*** value*
Age		25.10 ± 12.29	25.9 ± 13.43	0.84
Sex	Male	6 (46.1%)	6 (42.8%)	0.85
	Female	7 (53.9%)	8 (57.2%)	
Location of lesion	Upper extremities	10 (47.6%)	6 (30%)	0.33
	Lower extremities	7 (33.3%)	9 (45%)	
	Head and neck	4 (19%)	3 (15%)	
	Trunk	0	2 (10%)	

Values are mean ± SD and frequency (percentage) for continuous and categorical variables, ^*^Resulted from independent samples

*t*-test and chi-square test for continuous and categorical variables.

**Table 2:** Induration changes of the two groups during the study

**Group**	**Before**	**After 12 weeks**	**Mean difference**	***P*** -value*	***P*** -value**
Oral + liposomal group	2.90 ± 0.54 3 (2-4)	0.53 ± 0.77 0 (0-3)	-2.32 ± 0.82 -3 (-3,-1)	<0.001	0.09
Oral group	2.85 ± 0.67 3 (2-4)	1 ± 1.03 1 (0-3)	-1.85 ± 0.80 -2 (-3,0)	<0.001	

Values are mean ± SD and median (min-max).

^*^resulted from Wilcoxon signed-rank test for within-group comparisons and ^**^From Mann-Whitney U test for between-group analyses.

**Table 3:** Lesions size changes of the two groups during the study

**Group**	**Before**	**After 6** ^th^ ** week**	**After 12** ^th^ ** mouth**	***P*** **-value** **time**	***P*** **-value** **group**	***P*-value** **Time**+ **group**
Oral + liposomal	1.74 ± 0.58	0.34 ± 0.69	0.079 ± 34	<0.001	0.37	0.16
Oral group	1.65 ± 0.46	0.55 ± 0.58	0.27 ± 0.38	<0.001		

Values are mean ± SD, *P*-values resulted from linear mixed effect model.

**Table 4:** Improvement rate of the two groups during the study

**Improvement**	**After 6 week**		**After 12 week**	
	**Oral + liposomal group**	**Oral group**	**Oral + liposomal group**	**Oral group**
Slight	1 (4.8%)	6 (30%)	0	3 (15%)
Mild	0	0	0	0
Moderate	3 (14.3%)	4 (20%)	1 (4.7%)	4 (20%)
Significant	4 (19%)	1 (5%)	2 (9.4%)	0
Complete	11 (52.3%)	9 (45%)	18 (85.7%)	13 (65%)
*P*-Value	0.08		0.02	

## Discussion

The present study was the first clinical investigation concerning the anti-leishmaniasis effects in the combination of oral and topical liposomal azithromycin. Having positive results against *L. major*, the study showed that this combination is a safe alternative for CL. 

Azithromycin concentrates in tissues, especially in macrophages infected by *Leishmania* parasites, and can reach concentrations 100 to 200 times higher than in serum (5). 

Due to biodegradability, biocompatibility, non-toxic, and non-immunogenic nature, and capability of long-term sustained release of liposomes-loaded azithromycin, the combination of topical liposomal and oral azithromycin have been promising in the treatment of CL.

The liposomal drug delivery system is a great delivery system for the treatment of immune system diseases; thus, liposomes have been exploited for the delivery of antileishmanial agents (19).

The main important action is that liposomes passively target drugs to

Macrophages (20, 21)

Although the liposomal formulation of Amphotericin B has received FDA approval in the treatment of visceral leishmaniasis (22), it has been little efficacy against CL due to the weak therapeutic effect of Amphotericin B on CL’s parasite strains (23).

It seems that the use of liposomes has been neglected for CL. Not only could a liposomal formulation enhance the therapeutic effects of current chemotherapeutics, but also it might make the topical administration of a hydrophilic drug possible.

There is limited information on the application of liposomal paromomycin (24), Glucantime (12) and clarithromycin (25).

The use of Azithromycin for CL is incongruent, the reason of which may be the use of different definitions for the primary outcome measure or the variations in the method, dose and route of administration of azithromycin (oral *vs.* topical) (5, 8 and 9).

Oliveira *et al.* reported *in-vitro* antileishmania activity of azithromycin on promastigote and amastigote intracellular cultures against *L.* *amazonensis*, *L.* *braziliensis* and *L.* *chagasi*. They concluded that the azithromycin effect for the three species had been dose-dependent according to the results provided for *L. major *(8).

While Krolewiecki *et al.* demonstrated the azithromycin efficacy against *L. major in-vitro *and* in-vivo* in 2002 (5),

Two studies using azithromycin in the treatment of patients with old-world CL reported that azithromycin is not effective for the purpose, but the observations were not conclusive (10, 11). In the study of Momeni *et al.* (10) one of the main important limitations of this study is that patients were evaluated only at the end of treatment (21 days) and were not followed for a longer time, so the short duration of follow-up may have missed patients who would have been cured with a longer period of observation. In addition, although azithromycin decreases the number of CL parasites, the exact mechanism remains unclear (8).

In 2016, Rajabi *et al.* prepared liposomal formulations of azithromycin by the dehydration–rehydration vesicle (DRV) method, which caused the same efficacy as intralesional glucantime in the treatment of CL. These authors also showed that no serious drug side effects were observed (15).

The result of our study was compatible with the results of Rajabi *et al.* Also, our result showed a significant difference between the combination group and monotherapy after week 12 of the initiation of the study. For this reason, a more prolonged application of liposomal azithromycin may be more beneficial for the treatment of CL.

The present study has several limitations, including small sample size, a short observation period. Additionally, this was a pilot study, so it will be essential for future studies to establish suitable concentrations for treatment and the amount of medication for each time of treatment, and the treatment interval. The importance of this pilot study lies in observing the clinical effect of the combination of oral and topical liposomal azithromycin in the treatment of CL, which has not been studied before for this indication.

## Conclusion

The combination of oral and topical liposomal formulation of azithromycin is safe and effective for the treatment of old-world CL. In feature Large-sampled, appropriately designed, and randomized controlled clinical trials are required to evaluate therapeutic agents against CL.
